# Role of inflammatory caspases in a murine two-hit model of sepsis: analysis of immunosuppression and cognitive impairment

**DOI:** 10.1186/cc13004

**Published:** 2013-11-05

**Authors:** Mariana Gisely Amarante Teixeira da Cunha, Danielle Bastos de Albuquerque, Daiane Chaves, Silvio Caetano Alves Júnior, Flora Magno, Patrícia Torres Bozza, Fernando Augusto Bozza, Hugo Caire Castro-Faria-Neto, Rachel Novaes Gomes

**Affiliations:** 1 Laboratório de Imunofarmacologia, Fiocruz, RJ, Brazil; 2 Instituto de Pesquisa Evandro Chagas (IPEC), Fiocruz, RJ, Brazil

## Background

Morbidities associated with severe sepsis are serious problems for surviving patients, such as cognitive impairment and immunosuppression. The immunosuppression predisposes the patients to a second infection, which generally is fatal. Several studies have been made to understand the mediators involved with this immunosuppression associated with sepsis. Some data from the literature show that caspase-1 promotes activation of cytokines, such as IL-1β, and actions are inhibited by caspase-12. This study proposes to analyze the role of inflammatory caspases in immunosuppression and cognitive damage associated with a two-hit model of sepsis.

## Materials and methods

We submitted Swiss animals to the model of two hits of infection. The first hit was the CLP model and the second hit was intratracheal instillation of *Pseudomonas aeruginosa*. We analyze the mortality rate and the inflammatory profile of the animals submitted to the CLP model and the two-hit sepsis model. The cognition of the animals was tested by the passive avoidance test 15 and 21 days after the CLP and 21 days until 96 days after the two-hit sepsis model.

## Results

First we characterize the model and we observed a 30% survival rate of the CLP group in comparison with a 100% survival rate in the SHAM group. The high mortality of the CLP group was associated with hypoglycemia in the first 72 hours after the infection, increased neutrophil accumulation in the peritoneal cavity 6 and 24 hours after the CLP and an increase of inflammatory cytokines 6 hours after the CLP, such as CCL2, IL-1β and IL-10. The CLP group had a cognitive impairment 15 days after the CLP, but the memory was recovered 21 days after the infection. The CLP group was more susceptible to *P. aeruginosa *infection 21 days after the CLP, when we compare with the SHAM group. The CLP + *P. aeruginosa *group had a low count of neutrophils in BAL when compared with the SHAM + *P. aeruginosa *group. We observed a decrease in caspase-1 expression and an increased expression of caspase-12 in the lungs of the CLP + *P. aeruginosa *group. When we look to cognition, both the SHAM + *P. aeruginosa *and CLP + *P. aeruginosa *groups had cognitive impairment 21 days after the infection, and the cognitive impairment remained until 96 days in the SHAM + *P. aeruginosa *group after the infection, but the CLP + *P. aeruginosa *recovered the memory 96 days after the infection.

## Conclusions

Our preliminary results suggest that the immunosuppression associated with the CLP model (first hit) led to more susceptibility for survivor animals, which succumbed to a pneumonia model (second hit). We observed the involvement of inflammatory caspases in this immunosuppression phenomenon with a decrease of caspase-1 and an increase in caspase-12 expression. When we observed the cognitive function, we observed that the animals submitted to CLP had a cognitive impairment 15 days after the infection and the infection with *P. aeruginosa *induced a cognitive impairment until 96 days in both in groups. However, further studies should be made to confirm these results.

